# Development of an In Vitro Airway Epithelial–Endothelial Cell Culture Model on a Flexible Porous Poly(Trimethylene Carbonate) Membrane Based on Calu-3 Airway Epithelial Cells and Lung Microvascular Endothelial Cells

**DOI:** 10.3390/membranes11030197

**Published:** 2021-03-11

**Authors:** Thijs Pasman, Danielle Baptista, Sander van Riet, Roman K. Truckenmüller, Pieter S. Hiemstra, Robbert J. Rottier, Naomi M. Hamelmann, Jos M. J. Paulusse, Dimitrios Stamatialis, André A. Poot

**Affiliations:** 1 Technical Medical (TechMed) Centre, Department of Biomaterials Science and Technology, Faculty of Science and Technology, University of Twente, 7522 NB Enschede, The Netherlands; t.pasman@utwente.nl (T.P.); d.stamatialis@utwente.nl (D.S.); 2 Department of Instructive Biomaterials Engineering, MERLN Institute for Technology-Inspired Regenerative Medicine, Maastricht University, 6229 ER Maastricht, The Netherlands; danielle.baptista@maastrichtuniversity.nl (D.B.); r.truckenmuller@maastrichtuniversity.nl (R.K.T.); 3 Leiden University Medical Centre, Department of Pulmonology, Leiden University, 2300 RC Leiden, The Netherlands; s.van_riet@lumc.nl (S.v.R.); p.s.hiemstra@lumc.nl (P.S.H.); 4 Erasmus MC-Sophia Children’s Hospital, Departments of Pediatric Surgery and Cell Biology, 3000 CB Rotterdam, The Netherlands; r.rottier@erasmusmc.nl; 5 Department of Biomolecular NanoTechnology, Faculty of Science and Technology, University of Twente, 7522 NB Enschede, The Netherlands; n.m.hamelmann@utwente.nl (N.M.H.); j.m.j.paulusse@utwente.nl (J.M.J.P.)

**Keywords:** Calu-3 cells, LMVECs, PTMC, membranes, in vitro lung models, barrier function

## Abstract

Due to the continuing high impact of lung diseases on society and the emergence of new respiratory viruses, such as SARS-CoV-2, there is a great need for in vitro lung models that more accurately recapitulate the in vivo situation than current models based on lung epithelial cell cultures on stiff membranes. Therefore, we developed an in vitro airway epithelial–endothelial cell culture model based on Calu-3 human lung epithelial cells and human lung microvascular endothelial cells (LMVECs), cultured on opposite sides of flexible porous poly(trimethylene carbonate) (PTMC) membranes. Calu-3 cells, cultured for two weeks at an air–liquid interface (ALI), showed good expression of the tight junction (TJ) protein Zonula Occludens 1 (ZO-1). LMVECs cultured submerged for three weeks were CD31-positive, but the expression was diffuse and not localized at the cell membrane. Barrier functions of the Calu-3 cell cultures and the co-cultures with LMVECs were good, as determined by electrical resistance measurements and fluorescein isothiocyanate-dextran (FITC-dextran) permeability assays. Importantly, the Calu-3/LMVEC co-cultures showed better cell viability and barrier function than mono-cultures. Moreover, there was no evidence for epithelial- and endothelial-to-mesenchymal transition (EMT and EndoMT, respectively) based on staining for the mesenchymal markers vimentin and α-SMA, respectively. These results indicate the potential of this new airway epithelial–endothelial model for lung research. In addition, since the PTMC membrane is flexible, the model can be expanded by introducing cyclic stretch for enabling mechanical stimulation of the cells. Furthermore, the model can form the basis for biomimetic airway epithelial–endothelial and alveolar–endothelial models with primary lung epithelial cells.

## 1. Introduction

Lung diseases are still among the leading causes of death [1]. Moreover, the emergence of new respiratory viruses with a huge impact on society, such as SARS-CoV-2, further emphasizes the relevance of research on lung diseases. Unfortunately, in vitro human lung models frequently do not resemble the in vivo situation, and animal models are increasingly called into question due to ethical issues and generally low predictability for human diseases. Thus, there is an increasing need for the development of in vitro lung models that better mimic human lung tissue.

New in vitro models for airway and alveolar epithelial–endothelial cell interactions are very useful for research of lung diseases. Ideally, primary lung epithelial cells would be used for these models. However, this is often not feasible due to problems concerning the availability and culturing of these cells. For example, although advancements are made to make primary alveolar cells more accessible for research, their availability is limited and maintaining these cells in culture is difficult [2]. Thus, the majority of current lung models still relies on the use of cell lines. The widely used A549 alveolar cell line is less suited for studies on lung barriers since cultures hardly develop a barrier function. Therefore, the Calu-3 cell line, derived from human bronchial adenocarcinoma, has been used extensively in lung barrier models. These cells are regarded as a good model for lung airway epithelia since they can form a pseudostratified cell layer with a proper barrier function at the air–liquid interface (ALI) [3,4,5,6,7,8,9]. Moreover, Calu-3 cells are broadly available and easy to maintain. However, they are hardly applied in co-cultures together with endothelial cells, especially microvascular endothelial cells. As a result, many of the current Calu-3 lung barrier models lack the crosstalk between the lung epithelium and endothelium [10,11]. 

For a proper organ model, the support structure or membrane on which the cells are grown as well as the culture conditions, e.g., mechanical stimulation of the cells, should also be considered as these factors clearly affect the cells [12,13,14,15]. In some lungs-on-chips (LOCs), cells are cultured on a flexible and elastic membrane, typically made from poly(dimethylsiloxane) (PDMS) [12]. This enables mechanical stimulation of the cells by cyclic stretching or bending of the membrane. However, in many in vitro lung models, including LOCs, the cells are grown on stiff materials such as poly(ethylene terephthalate) (PET) [12], which has a much higher Young’s modulus than lung tissue (2–3 GPa [16] vs. 400 Pa or lower [17,18]). Since it is known that cells are affected by the stiffness of the material upon which they are grown [12], membranes made from softer, more flexible materials are preferable to better mimic the lungs. 

In this study, we aim to develop a new in vitro airway epithelial–endothelial model by co-culturing Calu-3 cells together with human lung microvascular endothelial cells (LMVECs) on flexible porous poly(trimethylene carbonate) (PTMC) membranes. We hypothesize that the lower stiffness of the PTMC membranes compared to PET membranes and the implementation of LMVECs will result in a lung epithelial–endothelial barrier model that would be an attractive alternative for existing models. The model could be utilized to study airway diseases that, for example, involve inflammation and damage of the lung, such as chronic obstructive pulmonary disease (COPD), asthma, bronchopulmonary dysplasia (BPD), and cystic fibrosis. Moreover, the effect of external factors (e.g., pollutants, pathogens, and medicines) on barrier function could be examined. Furthermore, the model could form the first step in developing biomimetic airway epithelial–endothelial and alveolar–endothelial models with primary lung epithelial cells. The latter would enable studies on alveolar diseases such as acute respiratory distress syndrome (ARDS) and study of the emphysema component of COPD. We chose the Calu-3 cells because of their robustness and proper barrier function of cell layers at ALI. The PTMC membranes have a Young’s modulus around 10 MPa, which is much lower than that of PET membranes, thus better mimicking the Young’s modulus of lung tissue. PTMC has been successfully used to fabricate films, membranes, and scaffolds for tissue engineering [19,20,21]. Moreover, PTMC-based films and scaffolds with suitable mechanical properties for cyclic stretch have been fabricated [20,22]. Here, we used PTMC membranes with various porosities, pore sizes, and water transport properties, which were developed earlier [23]. The PTMC membranes were either coated with levodopa (L-DOPA), a mixture of fibronectin, collagen I, and bovine serum albumin (FN/Col I/BSA) or L-DOPA followed by FN/Col I/BSA. L-DOPA is a precursor of dopamine and acts as a bioadhesive [24] that can stimulate the adsorption of proteins [25,26,27]. Chevtchik et al. have used L-DOPA as a bioadhesive to achieve optimal collagen IV coating of polymeric membranes for culturing kidney epithelial cells [26]. FN/Col I/BSA protein coatings have been successfully used as a coating for the culturing of primary lung epithelial cells [28]. The combination of the PTMC membrane and coating that resulted in the best adhesion and proliferation of Calu-3 cells was subsequently used to develop a co-culture of Calu-3 cells and LMVECs with the former cells cultured at ALI. Co- and mono-cultures were stained for Zonula Occludens 1 (ZO-1), a protein associated with tight junctions (TJs) and CD31, a marker for endothelial cells, and characterized in terms of cell viability and barrier function. Furthermore, the cultures were stained for mesenchymal markers to investigate epithelial- and endothelial-to-mesenchymal transition (EMT and EndoMT, respectively) and expression of these markers was compared to that of human vascular smooth muscle cells (SMCs). Results were compared to data from co- and mono-cultures on commercial PET membranes and discussed in view of the literature.

## 2. Materials and Methods

### 2.1. Materials

Acetic acid (cat. W200611), BSA (cat. A7030), calcein-AM (cat. 56496), Corning^®^ Transwell^®^ PET membrane cell culture inserts (cat. CLS3460), ethidium homodimer I (cat. E1903), fluorescein isothiocyanate-dextran (FITC-dextran) (cat. 46944), gelatin (cat. G1393), formaldehyde (FA) (cat. F1635), L-DOPA (cat. D9628), tris(hydroxymethyl) aminomethane (cat. 25,285-9), Triton^TM^ X-100 (cat. X100), and Tween^®^20 (cat. P9416) were all purchased from Sigma-Aldrich, St. Louis, MO, USA. Alexa fluor Donkey α Rabbit 568 antibody (cat. A10042), Alexa fluor Goat α Mouse 488 antibody (cat. A11029), and DAPI FluoroPure^TM^ (cat. D21490) were acquired from Life Technologies Corporation, Eugene, OR, USA. α-SMA antibody (cat. MA5-11547), CD31 antibody (cat. PA5-95186), vimentin antibody (cat. MA5-14564), and ZO-1 antibody (cat. 33-9100) were bought from Invitrogen, Rockford, IL, USA. DMEM (Dulbecco’s Modified Eagle’s Medium) (cat. 12491-015), fetal bovine serum (FBS) (cat. 10500-064), Glutamax^TM^ 100X (cat. 35050-061), and penicillin-streptomycin (P/S) (cat. 15140-122) were obtained from Gibco, Grand Island, NJ, USA. Dulbecco’s PBS without Ca^2+^/Mg^2+^ (DPBS) (cat. 14190-094) and 0.25% trypsin/EDTA (cat. 25200-072) were purchased from Gibco, Paisley, UK. Furthermore, we ordered Alexa Fluor™ 568 Phalloidin (Thermo Fisher Scientific, Waltham, MA, USA, cat. A12380), collagen I (Purecol) (Advanced Biomatrix, Carlsbad, CA, USA, cat. 5005), Dako fluorescent mounting medium (Dako North America, Carpinteria, CA, USA, cat. S3023), Eagle’s Minimum Essential Medium (EMEM) (Lonza Group Ltd., Basel, Switzerland, cat. BE12-662F), Falcon^®^ 12-well tissue culture-treated polystyrene (TCPS) permeable support companion plates (Corning GmbH, Wiesbaden, Germany, cat. 353503), fibronectin (EMD Millipore Corporation, Billerica, MA, USA, cat. FC010), Hoechst (Thermo Fisher Scientific, Waltham, MA, USA, cat. H1399), microvascular endothelial cell growth medium kit enhanced (Pelobiotech GmbH, Planegg/Martinsried, Germany, cat. PB-MH-100-4099), Nunc™ MicroWell™ 96-well, Nunclon Delta-treated, flat-bottom microplates (Thermo Fisher Scientific, Roskilde, Denmark, cat. 237107), and transforming growth factor β (TGF-β) (PeproTech Inc., Cranbury, NJ, USA, cat. 100-21).

### 2.2. Membranes and Coatings

#### 2.2.1. PTMC Membrane Fabrication

The synthesis of PTMC and fabrication of the membranes was described in detail previously [23]. Therefore, here we describe those only briefly. PTMC synthesis: PTMC was synthesized by ring-opening polymerization of trimethylene carbonate (TMC). After synthesis, the PTMC was purified and dried. Polymer dope composition: Polymer dopes for membrane fabrication contained PTMC of 600 kg/mol, poly(ethylene oxide) (PEO), pentaerythritol triacrylate (PETA), Irgacure 2959, hexanol, and chloroform. Hexanol was added as a non-solvent in a 1:0, 1:1, and 1:3 weight ratio of PTMC to hexanol. Membrane fabrication: Polymer dopes were cast on silicon wafers, and evaporation-induced phase separation (EIPS) was used to form the membranes, which are referred to as M0, M1, and M3 according to the weight ratio of PTMC to hexanol (i.e., 1:0, 1:1, and 1:3, respectively). All membranes were photo-crosslinked with UV-light, washed, and dried.

#### 2.2.2. Mounting of Membranes in Inserts

PTMC membranes in cell culture inserts: Circular samples with a diameter of 15 mm were punched from PTMC membranes. PET membranes of cell culture inserts were removed. The PTMC membrane samples were then secured to the underside of the inserts (substrate side [23] facing up) using poly(ether ether ketone) (PEEK) membrane rings (Figure S1 in Supplementary Material), provided by Ronald C. van Gaal (Department of Biomedical Engineering, Biomedical Materials and Biochemistry, Eindhoven University of Technology, Eindhoven, The Netherlands). Plate rings (Figure S1), also provided by Ronald C. van Gaal, were placed on top of wells of Falcon 12-well companion plates to elevate the inserts to allow cell culture medium to reach the underside of the inserts. The surface area of the PTMC membranes in the assembled inserts was the same as that of the inserts with the PET membranes (i.e., 1.12 cm^2^). Membrane and insert sterilization: Assembled inserts with PTMC membranes and inserts with PET membranes were kept in 70% ethanol for at least one hour. Afterwards, they were dried, washed, and left in culture plates in DPBS in a cell culture incubator.

#### 2.2.3. Membrane Coating

After sterilization, membrane samples either received no coating, an FN/Col I/BSA coating, an L-DOPA coating, or an L-DOPA coating followed by an FN/Col I/BSA coating. L-DOPA coating: The L-DOPA coating was performed following the protocol of Chevtchik et al. [26]. A 10 mmol/L Tris-buffer in MilliQ water was made. Acetic acid was added to lower the pH to 8.5. The buffer was sterilized by filtration through a 0.2 µm pore filter. Membrane samples in inserts were incubated in Tris-buffer for at least one hour. For the L-DOPA solution, a Tris-buffer with 2 mg/mL L-DOPA was sealed and left to stir for 45 min at 37 °C. Subsequently, the solution was immediately filtered through a 0.2 µm pore filter and added on top and underneath the membrane samples. Samples were then kept at 37 °C in an incubator for 20 h. Afterwards, the L-DOPA solution had turned almost completely black. Finally, samples were washed four times with DPBS and kept in DPBS at 37 °C in a cell culture incubator. While M1 and M3 PTMC membranes were white opaque before the L-DOPA coating, they were dark grey opaque afterwards, confirming that the L-DOPA coating was present on the membranes. The L-DOPA coating was also noticeable on M0 and PET membranes and TCPS, which all remained transparent. FN/Col I/BSA coating: A mixture consisting of fibronectin (5 µg/mL), collagen I (30 µg/mL), and BSA (10 µg/mL) in DPBS was sterile-filtered through a 0.2 µm pore filter and added to samples (both well and insert), which were then incubated for one hour at 37 °C. Afterwards, they were washed once with DPBS and kept at 37 °C in a cell culture incubator.

### 2.3. Cell Culture

#### 2.3.1. Cell Culture Media

Calu-3 medium: Cell culture medium used for the Calu-3 cells consisted of EMEM (88 vol%), FBS (10 vol%), P/S (1 vol%), and Glutamax^TM^ (1 vol%). LMVEC medium: A ‘microvascular endothelial cell growth medium kit enhanced’ was used for the culturing of LMVECs, according to the manufacturer’s instructions. Co-culture medium: Cell culture medium for the co-cultures consisted of Calu-3 (50 vol%) and LMVEC medium (50 vol%), as described above. SMC medium: Culture medium for the SMCs consisted of DMEM (88 vol%), FBS (10 vol%), P/S (1 vol%), and Glutamax^TM^ (1 vol%). For serum deprivation and stimulation of the SMCs with TGF-β, culture media were made consisting of DMEM (97.6 vol%), FBS (0.4 vol%), P/S (1 vol%), and Glutamax^TM^ (1 vol%), without and with TGF-β (5 ng/mL), respectively.

#### 2.3.2. Cells and General Cell Culturing

Calu-3 cells: Calu-3 cells (passage 8) were kindly provided by the department of Instructive Biomaterials Engineering, Maastricht University, Maastricht, The Netherlands. The cells were cultured in culture flasks with Calu-3 medium. At 70% confluence, cells were trypsinized and split before using them in plates and inserts between passage 11 and 16. LMVECs: LMVECs were bought from Pelobiotech GmbH, Planegg/Martinsried, Germany, cat. ACBRI 468. Cells were cultured in LMVEC medium in culture flasks coated with 0.1% gelatin. After reaching confluence, cells were trypsinized and split once before using them on inserts at passage 8. SMCs: SMCs from umbilical veins (passage 3) were received from Medical Spectrum Twente hospital, Enschede, The Netherlands, and cultured in SMC medium in culture flasks coated with 0.1% gelatin. At 70% confluence, cells were trypsinized and split before using them on inserts at passage 7. All cells were grown in a humid environment at 37 °C with 5% CO_2_. Cell culture medium was refreshed three times a week for all cells.

#### 2.3.3. Cell Seeding and Culturing on Inserts

Calu-3 cell culturing on PTMC membranes for membrane coating assessment: Calu-3 cells (passage 11) were seeded in cell culture inserts on M0, M1, M3, and PET membranes or in TCPS well plates with different coatings at a cell density of 120,000 cells/cm^2^. Cells were left to grow submerged for 8 days, after which those in the inserts were cultured at ALI by removing the Calu-3 medium from the insert. The Calu-3 medium was refreshed three times a week throughout the experiment. Calu-3 cells were also quickly washed three times a week with DPBS during ALI culturing to remove cell debris and mucus. After 2 weeks at ALI, the cells were fixed with 4% paraformaldehyde (PFA) and stained with DAPI and phalloidin (see Section 2.3.7 and Section 2.3.8). The cells on TCPS were cultured submerged for 23 days, and subsequently fixed and stained in the same manner.

**Scheme 1:**
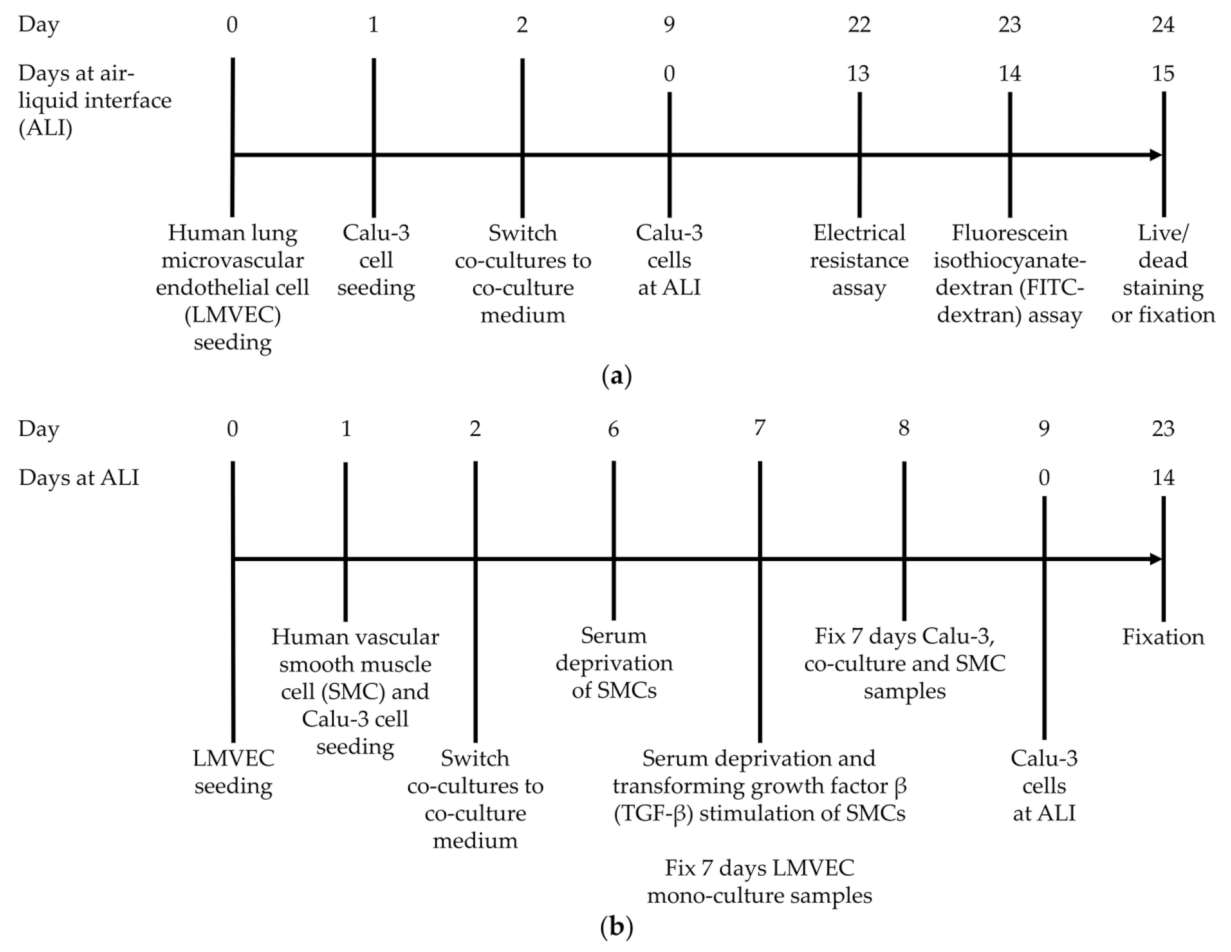
Timelines of cell culture experiments on poly(trimethylene carbonate) (PTMC) M3 and poly(ethylene terephthalate) (PET) membranes. Timelines were similar for co- and mono-cultures. (**a**) Timeline of cell culture experiments for tight junction protein Zonula Occludens 1 (ZO-1) and endothelial marker CD31 staining, live/dead staining, and barrier function assays. (**b**) Timeline of cell culture experiments for expression of epithelial-to-mesenchymal (EMT)/endothelial-to-mesenchymal (EndoMT) markers.

Cell culturing on M3 and PET membranes for ZO-1/CD31 staining and live/dead staining, and barrier function assays: LMVECs (passage 8) were seeded on the underside of cell culture inserts on L-DOPA+FN/Col I/BSA-coated M3 and PET membranes at a density of 80,000 cells/cm^2^ by flipping the cell culture inserts upside down. Cells were kept moist during attachment (approximately 2 h), after which the inserts were placed in 12-well plates in the normal orientation, and LMVEC medium was added to both the inserts and wells. The next day (day 1, see Scheme 1a), Calu-3 cells were seeded on the topside of selected membranes at 120,000 cells/cm^2^ in Calu-3 medium. For these co-cultures, cells were kept in their respective media. The next day (day 2), co-cultures were switched to co-culture medium, while mono-cultures were kept in their respective media. Samples with Calu-3 cells were transferred to ALI on day 9. Three times a week, the medium was refreshed, and Calu-3 cells were quickly washed with DPBS. Electrical resistance and FITC-dextran permeability assays were performed on day 22 and 23, respectively (13 and 14 days at ALI, respectively) (see Section 2.3.4 and Section 2.3.5). On day 24, the cells were fixed with 4% PFA or subjected to live/dead staining (see Section 2.3.7 and Section 2.3.8).

Cell culturing on M3 and PET membranes for EMT and EndoMT marker staining: For the expression of EMT/EndoMT markers, Calu-3 cells (passage 11) and LMVECs (passage 8) were seeded in the same manner as for the assessment of ZO-1/CD31 staining (see above) on L-DOPA+FN/Col I/BSA-coated M3 and PET membranes (Scheme 1b). Moreover, on day 1, SMCs (passage 7) were seeded at 10,000 cells/cm^2^ on L-DOPA+FN/Col I/BSA-coated M3 and PET membranes as positive controls for mesenchymal marker expression. SMCs were cultured in SMC medium. Calu-3/LMVEC co-cultures were switched to co-culture medium on day 2. On day 6, SMCs were serum-deprived by receiving SMC medium with 0.4 vol% FBS. On day 7, the SMCs were stimulated with 5 ng/mL TGF-β. Moreover, LMVEC mono-culture samples were fixed. On day 8, samples of Calu-3 mono-cultures, co-cultures, and all samples of the SMC cultures were fixed. At day 9, the remaining samples containing Calu-3 cells were transferred to ALI, as mentioned before. On day 23, all remaining Calu-3, LMVEC, and co-culture samples were fixed with 4% PFA (Section 2.3.7). Cell culture medium was refreshed three times a week, and during ALI culturing, the Calu-3 cells were quickly washed with DPBS three times a week as well.

#### 2.3.4. Electrical Resistance Measurements

12-well Transwell^®^ inserts with L-DOPA+FN/Col I/BSA-coated PET or M3 PTMC membranes were placed in a cellZscope system (nanoAnalytics GmbH, Münster, Germany). Fresh cell culture medium was added to the inserts. Preliminary measurements with a duration of three hours showed that the electrical resistance was stable during that time (data not shown). Nevertheless, measurement time in the case of membranes with cells was as short as possible (<1 h) to limit any potential influence of the assay on the cells. Electrical resistance data were collected by cellZscope software version 2.2.1 from nanoAnalytics (Münster, Germany). Data of two separate experiments were gathered, each experiment including samples of different membranes of each membrane type (total N ≥ 3 for membranes without cells, N ≥ 7 for samples with cells). For the type of reference insert, the option ‘Corning 12-well transparent (1.12 cm^2^)’ was chosen in the cellZscope software. The electrical resistance was measured from 1 Hz to 100 kHz.

#### 2.3.5. FITC-Dextran Permeability Assay

Sterile solutions of 1 mg/mL FITC-dextran (4 kg/mol) in cell culture medium were made. Membrane samples with or without cells were washed once with medium. Subsequently, 1.2 mL fresh medium was added underneath the inserts and 0.6 mL FITC-dextran solution was added to the inserts, on top of the membranes, for 60 min at 37 °C. Afterwards, samples of the medium underneath the inserts were taken in triplicate for analysis. The membrane samples with cells were then washed with medium and received fresh medium. Analysis was done by transferring the medium samples to black 96-well Microplates. Pure medium and a concentration series of FITC-dextran solutions were used as reference samples in each plate. The fluorescence was measured the same day by placing the plates in a Tecan Infinite M200 Pro^®^ plate reader with the Tecan i-control^TM^ software. The excitation and emission wavelengths were 490 nm and 520 nm, respectively. Data were collected from two separate experiments, each using samples of different membranes of each membrane type (total N = 4 for membranes without cells, N ≥ 9 for samples with cells). The data were corrected for the fluorescence values of the pure medium. A calibration curve was made to translate the fluorescence data to FITC-dextran concentrations. The apparent permeability coefficient (P_app_) in cm/s of FITC-dextran diffusion across the membrane and cell layers was then calculated using Equation (1) in which dQ is the accumulated FITC-dextran in mg in the acceptor compartment (i.e., the well), dt is the duration of the assay in seconds, C_0_ is the initial concentration of FITC-dextran in the donor compartment (the insert) in mg/cm^3^, and A is the surface area of the membrane, i.e., 1.12 cm^2^ for both the M3 and PET membranes.

(1) $$P_{\mathrm{app}}=\;\frac{\mathrm{dQ}/\mathrm{dt}}{C_{0}*A}$$

#### 2.3.6. Statistical Analysis

For statistical analysis of the electrical resistance and P_app_ data, a one-way ANOVA was performed using a Bonferroni post-hoc test with P < 0.05 in the Graphpad Prism 5 software (San Diego, CA, USA).

#### 2.3.7. Cell Fixation

Cells were quickly washed once with 37 °C DPBS, immediately followed by incubation in sterile 4 vol% PFA of 37 °C, both on top and underneath the inserts, for 45 min at 20 °C. Cells were washed three times with DPBS, left in DPBS, and stored at 4 °C.

#### 2.3.8. Immunocytochemistry

Calu-3 cell culturing, ZO-1/CD31 staining, and EMT and EndoMT markers: Fixed membrane samples with cells were incubated with a 0.1 vol% Triton^TM^ X-100 solution in DPBS for 10 min at 20 °C to permeabilize the cells. Subsequently, the samples were kept in a blocking solution, consisting of 5 wt% BSA, 2 vol% FBS, and 0.1 vol% Tween^®^20 in DPBS, for at least 60 min. Calu-3 samples for the assessment of the polymer membrane coating were incubated with a phalloidin solution (1:200 dilution in DPBS) for 30 min at 20 °C to stain F-actin. Afterwards, these samples were incubated with a DAPI solution (see below). All other samples were incubated overnight at 4 °C with either a ZO-1 or α-SMA antibody (1:300 and 1:200 dilution in DPBS, respectively). The next day, samples were incubated with an Alexa fluor Goat α Mouse 488 solution (1:200 dilution in DPBS) for 2 h at 20 °C. Subsequently, samples were incubated overnight at 4 °C with either a CD31 or vimentin antibody (both 1:500 dilution in DPBS). The next day, samples were incubated with an Alexa fluor Donkey α Rabbit 568 antibody (1:500 dilution in DPBS) for 2 h at 20 °C. Samples were then incubated with a DAPI solution (300 nM in DPBS) for 20 min at 20 °C. All incubation steps were done in the dark, and all samples were washed at least 2 times with DPBS between all steps. Afterwards, Calu-3 samples were mounted on microscopy slides with mounting medium and then imaged.

Calu-3 samples for the assessment of the polymer membrane coating were imaged with an EVOS FL AMF4300 microscope (Advanced Microscopy Group, Bothell, WA, USA), whereas images of the cellular ZO-1/CD31 staining and EMT/EndoMT markers were made with a Leica TCS SP5 confocal microscope with the Leica Application Suite software (Leica Microsystems Inc., Buffalo Grove, IL, USA). The Calu-3 samples for assessment of the membrane coating were taken from one experiment (N = 1), and samples for the ZO-1/CD31 staining originated from different membranes of two separate experiments (total N = 3 for each of the stainings). Samples for the staining of EMT and EndoMT markers came from different membranes of one experiment (N = 3).

Live/dead staining for cell viability: A solution containing 2 µM calcein, 4 µM ethidium homodimer I, and 16 µM Hoechst in cell culture medium was prepared. The unfixed cells were sensitive to the staining procedure, especially to washing with DPBS, which affected their morphology or even adhesion. Therefore, samples were washed once with their respective culture medium instead of DPBS. Cells received the staining solution and were incubated for 20 min, after which the samples were washed with medium, and the membranes were placed on microscopy slides. Samples were immediately imaged on a Nikon Eclipse 400 microscope (Nikon, Tokyo, Japan) with the Nikon NIS-Elements Imaging software (version 4.51.01). Samples were taken from different membranes of two separate experiments (total N = 3).

## 3. Results and Discussion

### 3.1. Increased Calu-3 Cell Numbers on PTMC Membranes With L-DOPA and FN/Col I/BSA Coating

**Figure 1:**
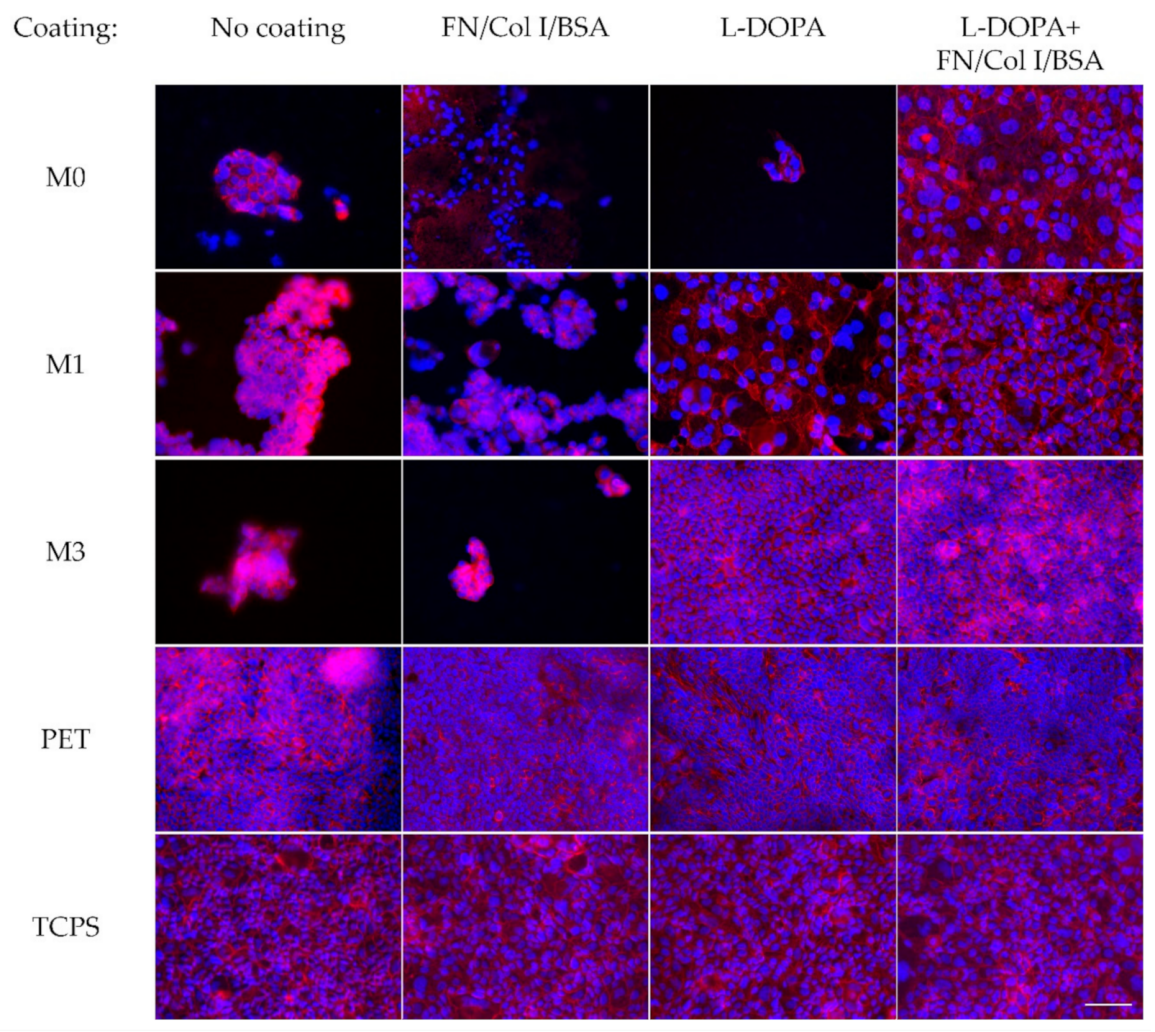
Cell nucleus (DAPI, blue) and F-actin (red) staining of Calu-3 cells grown for 8 days submerged and 2 weeks at air–liquid interface (ALI) on poly(trimethylene carbonate) (PTMC) and poly(ethylene terephthalate) (PET) membranes with different coatings. The latter membranes as well as tissue culture-treated polystyrene (TCPS) wells in which the cells were cultured submerged for 23 days, were used as references. Magnification: 200×. Scale bar: 100 µm, applies to all images. Samples originated from one experiment (N = 1).

Adhesion of Calu-3 cells on PTMC M0, M1, and M3 membranes was assessed by staining their nuclei (Figure 1). The PTMC membranes had diverse properties, as described in a previous paper [23], most notably concerning their porosity and water transport. Briefly, M0 membranes were non-porous and non-permeable. M1 and M3 membranes had porosities of 21% and 42%, and water permeances of 17,000 and 41,000 L/(m^2^ h bar), respectively. Furthermore, pore sizes of M1 and M3 membranes were 5–8 and 7–8 µm, respectively. Different coatings were applied on these PTMC membranes, as well as on PET membranes with 0.4 µm pores and on TCPS cell culture plates. Calu-3 cells were stained after eight days of submerged culture and two weeks of culture at ALI on PTMC and PET membranes, and after 23 days of submerged culture on TCPS.

M0, M1, and M3 membranes without coating or with a coating of FN/Col I/BSA contained clusters of Calu-3 cells (Figure 1). Cells grew poorly on all M0 membranes, as was expected since these membranes were not porous. The number of cells on the M0 membrane with L-DOPA+FN/Col I/BSA coating was higher than on the other M0 membranes, but cell density was still low, and the cells and nuclei were relatively large. Cell adhesion on M1 membranes with L-DOPA or L-DOPA+FN/Col I/BSA was better than on the other M1 membranes. On the M1 membrane coated with L-DOPA+FN/Col I/BSA, the cell density was higher than on the M1 membrane provided with L-DOPA coating alone. On the latter membrane, cells were not confluent, and both the cells and their nuclei were large. The positive effect of L-DOPA coating on Calu-3 cell adhesion and proliferation was most noticeable with M3 membranes coated with L-DOPA or L-DOPA+FN/Col I/BSA, which were completely covered with cells, especially for the latter coating. Moreover, these cell layers resembled those on PET membranes, both in cell density and size of the cells and their nuclei. Cell layers on PET membranes were all confluent, dense, and there was no difference in the size of the nuclei between the coating conditions. Cell coverage on TCPS was also homogeneous regardless of the coating, and samples were quite confluent. However, cell numbers were lower on TCPS than on PET since the cells on TCPS were relatively large. Moreover, some of the cells lifted off the TCPS during culturing.

The low adhesion and growth of Calu-3 cells on the non-coated and FN/Col I/BSA-coated PTMC membranes is probably caused by PEO in the membranes, which is non-cell adhesive [29,30,31] and inhibits protein adsorption on surfaces [31]. In contrast, L-DOPA stimulates adsorption of proteins [25,26]. Thus, adsorption of proteins from the FN/Col I/BSA coating or the cell culture medium was most probably improved on the membranes coated with L-DOPA, which resulted in a higher and more homogeneous cell coverage on these membranes.

The apparently large size of Calu-3 cells and their nuclei on some M0 and M1 membranes could be due to swelling or flattening of the cells and was probably caused by an external stimulus or lack thereof. For example, hyperoxia has been found to cause swelling of the nuclei of Calu-3 cells [32]. Although our cells were not cultured in hyperoxia, the surviving cells on the non-porous M0 membranes must have experienced a lot of stress due to a lack of nutrients. In addition, in our cultures, large nuclei and cells were not seen in dense monolayers or clusters. Thus, cells on the M0 and some of the M1 membranes probably experienced a lack of nutrients and/or insufficient cell-cell contact. This probably resulted in swelling or flattening of the cells (and nuclei), the latter as an attempt to receive more nutrients or improve cell-cell contact. Cells which received insufficient nutrients were probably only capable of staying adhered to the membranes because of the robustness of the cells.

Calu-3 cells developed cell layers on both M1 and M3 membranes with the L-DOPA+FN/Col I/BSA coating. Cultures on M3 membranes had higher cell densities than the cultures on M1 membranes, which is probably due to the higher permeability of the M3 membranes compared to the M1 membranes. The PET membranes performed better than the M1 membranes, despite having lower water permeance than the latter (i.e., 3000 vs. 17,000 L/(m^2^ h bar), respectively) [23]. Probably, the pore distribution of PET membranes was more homogeneous than that of M1 membranes. As a result, cell layers on the M1 membranes were less confluent than on PET membranes. In contrast, the cultures on M3 membranes closely resembled the cultures on PET membranes. Therefore, we continued with M3 membranes and kept PET membranes as a reference. Moreover, all membranes were coated with L-DOPA+FN/Col I/BSA in further experiments.

### 3.2. Calu-3 Cells Express ZO-1, and LMVECs Show CD31 Expression

**Figure 2:**
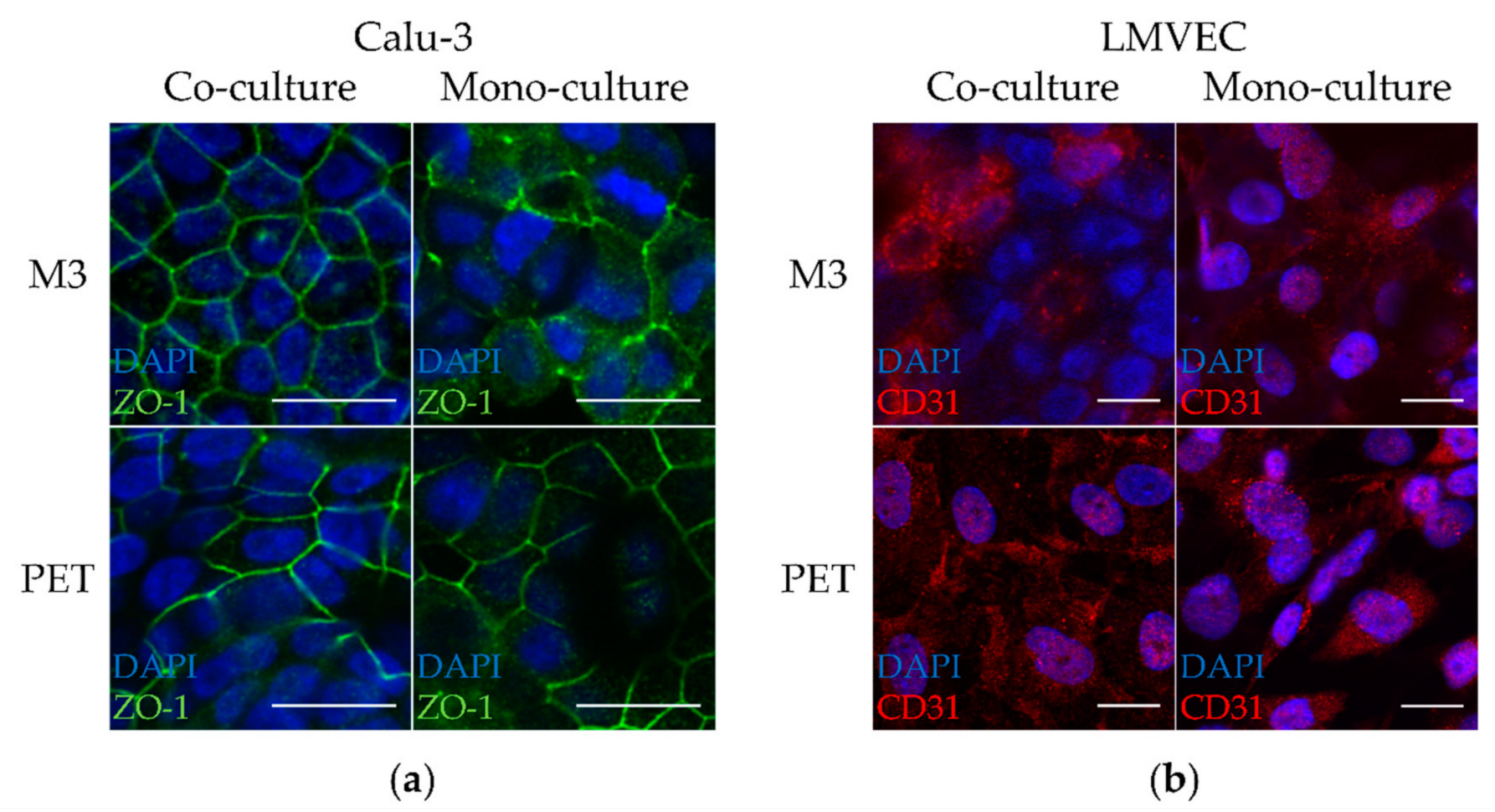
Staining of the tight junction protein Zonula Occludens 1 (ZO-1) and the endothelial marker CD31 of co- and mono-cultures of Calu-3 cells and human lung microvascular endothelial cells (LMVECs) in inserts on M3 membranes. Cells grown on inserts with PET membranes were used as controls. (**a**) Calu-3 cells were cultured and submerged for 8 days, followed by 2 weeks of culture at ALI. Cells are stained for the cell nucleus (blue) and tight junctions (ZO-1, green), magnification: 3200×, scale bar: 20 µm. (**b**) LMVECs after 3 weeks of submerged culture. Cells are stained for the cell nucleus (blue) and CD31 (red), magnification: 1600×, scale bar: 20 µm. Images are representative for observations of samples from two independent experiments (total N = 3).

Co- and mono-cultures of Calu-3 cells and LMVECs on M3 and PET membranes were stained for ZO-1 and CD31 (Figure 2). Cells were cultured submerged for three weeks in the case of the LMVECs or eight days submerged, followed by two weeks at ALI in the case of the Calu-3 cells.

Calu-3 cells formed confluent cell layers on the M3 membranes and had a typical cobblestone phenotype [33] with generally little cytoplasm (Figure 2a). Cells showed good ZO-1 expression. ZO-1 expression in Calu-3 cells of the co-cultures on PTMC and PET membranes appeared to be somewhat more localized at the cell membrane compared to that in Calu-3 mono-cultures. This requires confirmation in future studies using more objective, quantitative approaches. Nevertheless, these observations may be explained by interactions mediated by soluble factors, since such factors secreted by human umbilical vein endothelial cells (HUVECs) were reported to increase the production of the TJ-associated protein occludin in human bronchial epithelial-derived cells (16HBE 14o-cells) [11]. Cultures of Calu-3 cells on PET membranes were very similar to those on M3 membranes in terms of nucleus size, and the shape, size, and density of cells (Figure 2a).

LMVECs on the M3 membranes were stained for the protein CD31 (Figure 2b). Cells adhered well to the M3 membranes and expressed CD31 but did not form a confluent monolayer. The expression of CD31 was diffuse without a pronounced staining at the cell membrane. Cell densities and CD31 expression were similar between LMVECs in co- and mono-cultures and did not differ between M3 and PET membranes. Weppler et al. found that mono-cultures of HUVECs cultured on porous membranes showed a pronounced CD31 expression at their cell membranes, while the expression was more diffuse in co-cultures with lung epithelial cells, including Calu-3 cells, on the other side of porous membranes [34]. Moreover, while cell layers of HUVEC mono-cultures were confluent, HUVECs in co-cultures did not reach full confluency. These results are in line with the dispersed CD31 and incomplete confluency of LMVECs in our co-cultures. However, this does not explain the similar results seen in the LMVEC mono-cultures. The diffuse expression of CD31 and lack of localization at the membrane in LMVECs of both co- and mono-cultures could be an indication of EndoMT. However, we did not find signs of EndoMT in terms of α-SMA expression (see Section 3.5 on EMT and EndoMT marker expression).

### 3.3. Little Cell Death of Calu-3 Cells and LMVECs Cultured on PTMC Membranes

**Figure 3:**
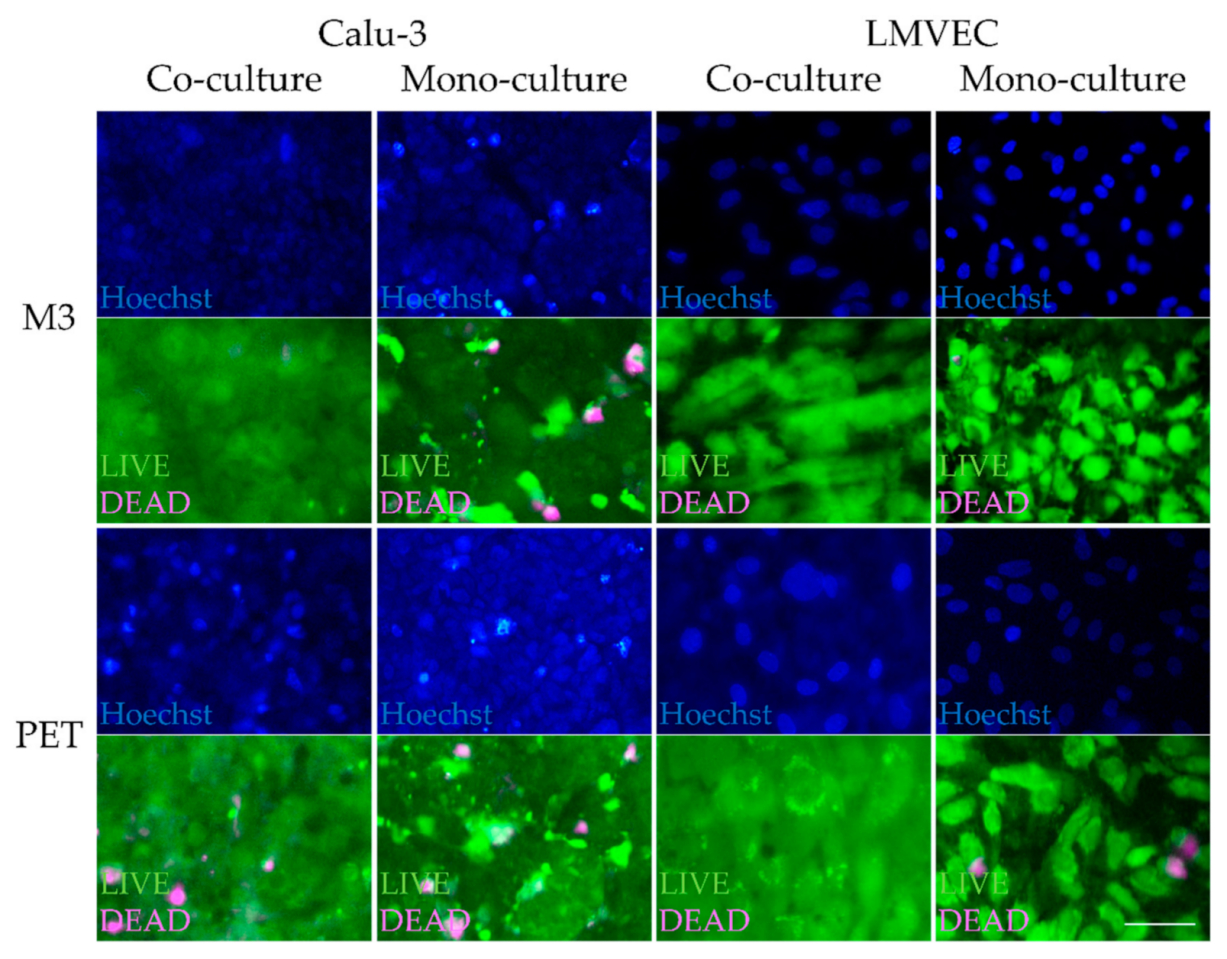
Live/dead staining of co- and mono-cultures of Calu-3 cells and LMVECs on M3 membranes. Cells on inserts with PET membranes served as controls. Calu-3 cells were cultured submerged for 8 days, after which they were cultured at ALI for 2 weeks. LMVECs were cultured submerged throughout the experiment. Images show the cell nuclei (Hoechst: blue), and the living (calcein: green) and dead cells (ethidium homodimer I: magenta). Magnification: 400×. Scale bar: 50 µm, applies to all images. Images are representative for observations of samples from two independent experiments (total N = 3).

The viability of the co- and mono-cultures of Calu-3 cells and LMVECs on M3 and PET membranes after three weeks of submerged/ALI culture was determined by a live/dead staining (Figure 3). Cell viability was high for both Calu-3 cells and LMVECs on M3 membranes with limited cell death, as shown by the green calcein and magenta ethidium homodimer I signals, respectively. While the level of calcein signal differed between individual Calu-3 cells in mono-cultures, Calu-3 cells in co-cultures showed a very consistent and overall higher calcein signal. Moreover, Calu-3 cell death was lower in co-cultures than mono-cultures. There was little difference in the level of calcein signal between individual LMVECs of mono-cultures on M3 membranes. This was also found for LMVECs of co-cultures, but the calcein signal was more diffuse than in mono-cultures. The LMVECs in mono-cultures probably contracted somewhat during the assay since they were sensitive to the staining procedure, while those in co-cultures remained spread. Prior to the assay, LMVECs had probably attained better cell adhesion in co-cultures than in mono-cultures due to soluble factors in the co-cultures.

In general, the calcein and ethidium homodimer I signals of cultures on PET membranes were similar to those on M3 membranes (Figure 3). LMVECs of co-cultures on PET membranes showed a more dispersed calcein signal than those on M3 membranes or the LMVEC mono-cultures. This dispersed calcein signal was probably present because calcein-positive Calu-3 cells on the other side of the transparent PET membranes were also visible in the case of co-cultures.

These data indicate a good cell viability on M3 membranes, which was better in co-cultures than in mono-cultures. As described before, it is known that there is paracrine crosstalk between the lung epithelium and endothelium [10,11]. Probably, the lower Calu-3 cell death in our co-cultures compared to the mono-cultures was caused by soluble factors.

### 3.4. High Electrical Resistance and Low Permeability for FITC-Dextran of Calu-3 Cell and LMVEC Co-Cultures on PTMC Membranes

**Figure 4:**
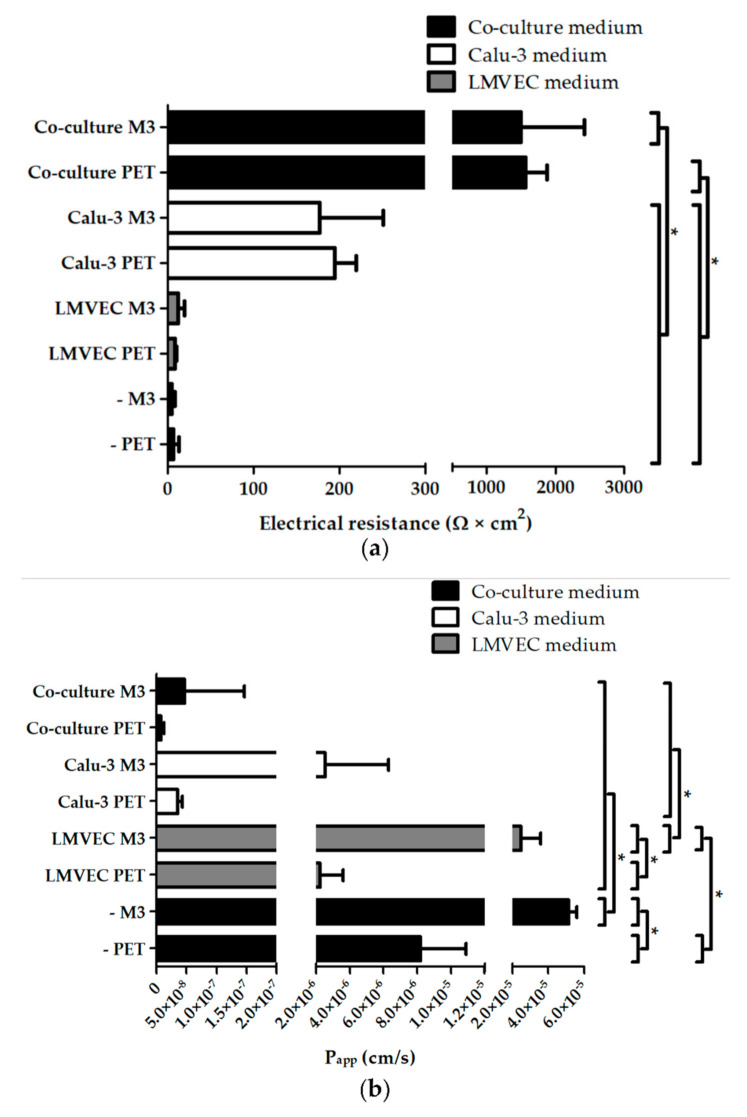
Electrical resistance and P_app_ of fluorescein isothiocyanate-dextran (FITC-dextran) diffusion across co- and mono-cultures of Calu-3 cells and LMVECs on M3 and PET membranes. Calu-3 cells were cultured submerged for 8 days, followed by 2 weeks at ALI. (**a**) Electrical resistance and (**b**) FITC-dextran permeability assays were performed on inserts with M3 or PET membranes containing either no cells, Calu-3 cells, LMVECs, or co-cultures. Capped lines depict significant differences, *P* < 0.05. Samples originated from two independent experiments (total N ≥ 3 for membranes without cells and N ≥ 7 for samples with cells). The electrical resistance and FITC-dextran permeability of membranes without cells in Calu-3 and LMVEC medium (data not shown) were similar to those of respective samples in co-culture medium.

The barrier function of the Calu-3 and LMVEC co- and mono-cultures on M3 and PET membranes was assessed by the electrical resistance and apparent permeability of 4 kg/mol FITC-dextran (Figure 4). M3 and PET membranes without cells were included as controls.

Co-cultures had a good barrier function on M3 membranes, as indicated by a high electrical resistance and low permeability for FITC-dextran, i.e., 1490 Ω cm^2^ and 4.7 × 10^−8^ cm/s, respectively (Figure 4). Co-cultures on M3 membranes had much higher electrical resistance than Calu-3 cell and LMVEC mono-cultures and M3 membranes without cells, i.e., 1490 vs. 177, 13, and 6 Ω cm^2^, respectively (Figure 4a). The FITC-dextran permeability of co-cultures was lower than that of LMVEC mono-cultures and M3 membranes without cells, i.e., 4.7 × 10^−8^ vs. 2.5 × 10^−5^ and 5.1 × 10^−5^ cm/s, respectively (Figure 4b). Statistical analysis without the latter two conditions showed that FITC-dextran permeability of co-cultures was also significantly lower than that of Calu-3 mono-cultures on M3 membranes (2.5 × 10^−6^ cm/s).

Calu-3 mono-cultures on M3 membranes had a significantly better barrier function in terms of FITC-dextran permeability than LMVEC mono-cultures and M3 membranes without cells. Moreover, statistical analysis of the electrical resistance without the very high values of the co-cultures showed a higher electrical resistance of the Calu-3 mono-cultures than of the LMVEC mono-cultures and M3 membranes without cells. In general, LMVEC mono-cultures had a very poor barrier function. Although they had a lower permeability for FITC-dextran than M3 membranes without cells, the electrical resistance was similar in both cases. The difference between the assays can be explained by the much larger size of FITC-dextran (4 kg/mol) used in the permeability assay, compared to the ions and charged molecules forming an electrical current in the electrical resistance assay.

There were no differences in electrical resistance between cell cultures on M3 and PET membranes. This indicates that the higher water permeance of the M3 membranes compared to the PET membranes, as determined by us before [23], did not influence the formation of cell layers on these membranes. This fits well with the immunofluorescence data (Figure 2). Co-cultures on M3 and PET membranes showed similar results in the FITC-dextran permeability assay. However, the permeability for FITC-dextran of Calu-3 cell and LMVEC mono-cultures was higher on M3 membranes than on PET membranes. This is consistent with the higher FITC-dextran permeability of bare M3 membranes compared to PET membranes without cells, which is in agreement with their water permeance [23]. These data indicate that diffusion of the FITC-dextran molecules was already hampered by the relatively low permeance of the PET membranes, while the ions and small molecules involved in the electrical current were not influenced. Consequently, there were differences between mono-cultures on M3 and PET membranes in terms of permeability for FITC-dextran, but not in terms of electrical resistance.

In other studies, with Calu-3 cell mono-cultures, similar FITC-dextran permeabilities were reported as in our study, indicating a good barrier function of our Calu-3 cell mono-cultures [7,8]. Moreover, the electrical resistance data of our Calu-3 cell mono-cultures (177 Ω cm^2^ and 195 Ω cm^2^ on M3 and PET membranes, respectively) are in line with literature [4,6,7,8,9,35,36]. Values of 50–2500 Ω cm^2^ have been reported for Calu-3 cell layers, which were dependent on factors such as culture conditions (e.g., submerged or ALI culture) and measuring equipment [4,6,7,8,9,35,36]. The poor barrier function of LMVEC mono-cultures found by us has also been reported in other studies using endothelial cells [35,36,37], including LMVECs [36].

Our data showed that our system has better barrier function for the co-cultures than the mono-cultures, which is in agreement with electrical resistance data of co- and mono-cultures of Calu-3 and endothelial cells using different support structures and membranes reported in literature [35,36]. These studies reported electrical resistances of 1352 [35] and >1000 Ω cm^2^ [36] for the co-cultures, similar to that of our co-cultures on M3 and PET membranes (1490 and 1560 Ω cm^2^, respectively). However, the difference between co-cultures and Calu-3 cell mono-cultures in those studies was smaller than the difference found by us, because of the higher electrical resistance of Calu-3 cell mono-cultures in the former studies [35,36] compared to our study. Formally, we cannot exclude that the different medium used for the co- and mono-cultures has contributed to the difference between them. However, since the co-culture medium was a mix of the epithelial and endothelial medium, this unlikely fully explains the difference.

The better barrier function of our co-cultures compared to the mono-cultures indicates a synergistic interaction between the Calu-3 cells and LMVECs in co-cultures. Based on other reports in the literature, this effect is most probably mediated by soluble factors secreted by the cultured cells rather than epithelial–endothelial cell-cell contact [10,11]. Bärnthaler et al. showed that A549 alveolar epithelial cells secreted factors which improved the barrier function of LMVECs [10]. In contrast, Chowdhury et al. found a paracrine effect of HUVECs on 16HBE 14o-epithelial cells, but not the other way around [11]. These data illustrate a complex crosstalk between epithelium and endothelium, which is not fully understood yet and probably depends on multiple factors. These studies support our observations, which emphasize the need for a co-culture of lung and endothelial cells to better mimic lung epithelial–endothelial barriers.

### 3.5. Low Vimentin Expression in Calu-3 Cells and Low α-SMA Expression in LMVECs of Co-Cultures on PTMC Membranes

**Figure 5:**
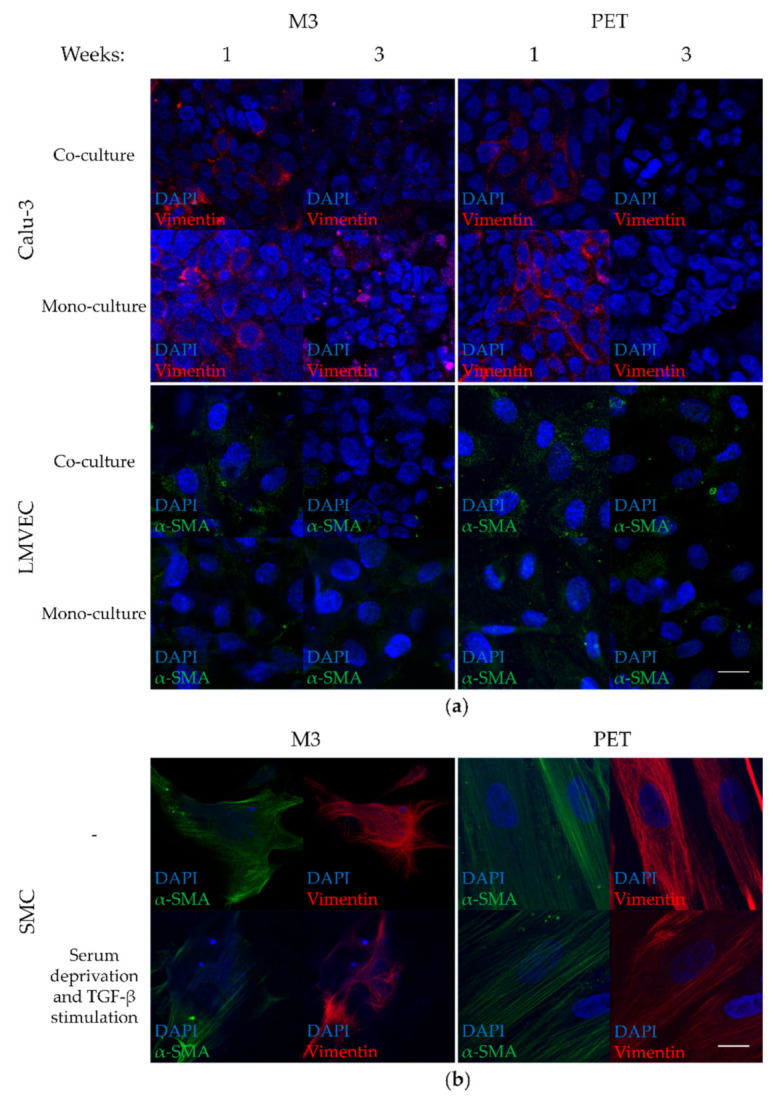
Staining for epithelial-to-mesenchymal (EMT) and endothelial-to-mesenchymal (EndoMT) markers of cells on M3 and PET membranes. (**a**) Co- and mono-cultures of Calu-3 cells and LMVECs were cultured in inserts on M3 or PET membranes. Cells were cultured submerged for 1 week, after which they were either fixed or cultured for 2 additional weeks (at ALI in the case of Calu-3 cells) and fixed. Calu-3 cells were stained for the cell nucleus (blue) and vimentin (red). LMVECs were stained for the cell nucleus (blue) and α-SMA (green). Magnification: 1600×. Scale bar: 20 µm, applies to all images. (**b**) SMCs were stained as positive controls for mesenchymal marker expression. They were grown submerged for 5 days on M3 or PET membranes. Some cells were then serum-deprived and stimulated with transforming growth factor β (TGF-β). All samples containing human smooth muscle cells (SMCs) were fixed on day 8. SMCs were stained for the cell nucleus (blue), α-SMA (green), and vimentin (red). Magnification: 1600×. Scale bar: 20 µm, applies to all images. Images are representative for observations of multiple samples from one experiment (total N = 3).

The development of EMT and EndoMT of the cells in the model was assessed by staining Calu-3 cells, LMVECs, and SMCs on M3 and PET membranes for EMT and EndoMT markers (Figure 5). Expression of vimentin, an intermediate filament protein in mesenchymal cells such as fibroblasts and SMCs, and a late marker for advanced stages of EMT, was used to investigate EMT in the Calu-3 cells (Figure 5a). α-SMA, a protein abundantly present in microfilaments of myofibroblasts, SMCs, and other mesenchymal cells, was investigated to assess EndoMT in the LMVECs (Figure 5a). SMCs served as positive controls for mesenchymal marker expression. Serum deprivation and TGF-β stimulation were used to upregulate the expression of vimentin and α-SMA in SMCs.

Calu-3 cells in co- and mono-cultures on M3 membranes showed little expression of vimentin after seven days of submerged culture and two weeks of additional culture at ALI, i.e., three weeks in total (Figure 5a). We did not observe differences in expression of vimentin between cells cultured on PTMC and PET membranes, suggesting that possible development of EMT was not associated with culture of Calu-3 cells on PTMC membranes. Expression of vimentin was lower after ALI culture than after seven days of submerged culture. As Calu-3 cells acquire several characteristics similar to native epithelium when cultured at ALI [7,8], the additional culture at ALI probably led to similar changes in our cultures, including a decrease in vimentin expression. LMVECs in co- and mono-cultures on M3 membranes showed a faint expression of α-SMA evenly throughout the cytoplasm, which did not change between one and three weeks of culture, and was comparable to that of LMVECs on PET membranes (Figure 5a).

Unstimulated and stimulated SMCs on M3 and PET membranes expressed high amounts of vimentin and α-SMA, and both proteins showed a filamentous organization (Figure 5b). SMCs on M3 membranes were smaller and less spindle-like, with less filamentous organization of vimentin and α-SMA and less distinct cell nuclei than SMCs on PET membranes. This can be explained by the lower stiffness of M3 compared to PET membranes, in agreement with literature [38]. This indicates that, even without external mechanical actuation of the flexible membrane, the lower stiffness of the PTMC membrane can already invoke different cell responses than the PET membranes.

The expression of vimentin and α-SMA was much higher in SMCs than in any of the Calu-3 and LMVEC co- and mono-cultures. Moreover, vimentin and α-SMA in Calu-3 cells and LMVECs lacked the filamentous organization found in the SMCs (Figure 5a,b). Togami et al. reported that Calu-3 cells express some vimentin under baseline conditions without a loss of epithelial characteristics or signs of attaining a migratory state [39]. This was indicated by a high expression of the epithelial marker E-cadherin and TJ-related Claudin 1, 3, and 5 in the cells. Francescone et al. showed that human microvascular endothelial cells, which expressed typical endothelial markers and had the capacity for network formation, expressed a small amount of α-SMA by default [40]. Thus, using vimentin as a late marker for advanced stages of EMT, we did not obtain evidence for development of EMT in Calu-3 cells as a result of culturing on the membranes. Similarly, using α-SMA as a marker for EndoMT, there were no signs of EndoMT of the LMVECs in our cultures.

### 3.6. Calu-3 Cell and LMVEC Co-Cultures on PTMC Membranes Form a Functional Barrier Model That Can Be Expanded

Our in vitro airway epithelial–endothelial model consisting of Calu-3 cells and LMVECs on flexible porous PTMC membranes showed good ZO-1 staining in Calu-3 cells, and LMVECs were CD31-positive. Cells had high viability, and barrier function was comparable to similar cultures in literature, which confirmed the functionality of the presented model. Moreover, it offers possibilities for extension. Films and scaffolds based on crosslinked PTMC networks have shown mechanical properties suitable for cyclic stretch [20,22]. This most probably also holds for the M3 membranes, as they comprise crosslinked PTMC networks as well [23]. The presented airway epithelial–endothelial model could be mounted in a LOC and subjected to cyclic stretch and/or membrane bending to mimic the dynamic environment in a breathing lung. Moreover, the M3 membranes were formed by EIPS, a method that could be used in conjunction with micro-moulding [41,42,43]. In this way, microchannel-like or semi-spherical microstructures could be introduced in the PTMC membranes to better resemble the bronchioles or alveoli, respectively [12,44]. Such microstructures can influence cell characteristics like orientation, proliferation, and differentiation [12,44]. Moreover, as mentioned, there is a need for both airway epithelial–endothelial and alveolar–endothelial models. This study focused on the former, but also forms a basis for expansion towards alveolar models. The presence of multiple relevant lung cell types is essential for development of more advanced lung barrier models. Besides epithelial and endothelial cells, as presented in this study, future models should also focus on implementation of fibroblasts and immune cells.

## 4. Conclusions—Outlook

This study combines for the first time Calu-3 cells with LMVECs on flexible porous PTMC membranes to produce an airway epithelial–endothelial model. The model displays important characteristics such as ZO-1 staining by the Calu-3 cells, CD31-positive LMVECs, high cell viability, and a comparable barrier function to similar co-cultures in literature. Upon culturing on PTMC membranes, Calu-3 cells and LMVECs showed no increased expression of the mesenchymal markers vimentin and α-SMA, respectively. The data also illustrate that implementation of endothelial cells provides multiple benefits, such as an improved barrier function compared to mono-cultures. This is probably caused by soluble factors, similar to the paracrine crosstalk between epithelial and endothelial cells. Future implementation of our model in LOCs that provide cyclic stretch and/or bending of the membrane would allow mechanical stimulation of the cells. This enables studying the impact of the mechanical forces of breathing, which cannot be achieved with PET membranes. These characteristics make this airway epithelial–endothelial model of Calu-3 cells and LMVECs on PTMC membranes a possible alternative model for lung research, as well as a foundation for more advanced models.

## Data Availability

The data presented in this study are available on request from the corresponding author.

## Supplementary Materials

The following are available online at https://www.mdpi.com/2077-0375/11/3/197/s1, Figure S1: Cell culture inserts for the poly(trimethylene carbonate) (PTMC) membranes.
