# Supplementary material for: Development of an In Vitro Airway Epithelial–Endothelial Cell Culture Model on a Flexible Porous Poly(Trimethylene Carbonate) Membrane Based on Calu-3 Airway Epithelial Cells and Lung Microvascular Endothelial Cells
Source: Membranes (Basel). 2021 Mar 11;11(3):197. doi: 10.3390/membranes11030197 (PMC8001677; doi:10.3390/membranes11030197)
Supplement: Supplementary file 1 [file membranes-11-00197-s001.pdf]

membranes

Supplementary Material: Development of an In Vitro Airway Epithelial-Endothelial Cell Culture Model on a Flexible Porous Poly(Trimethylene Carbonate) Membrane Based on Calu-3 Airway Epithelial Cells and Lung Microvascular Endothelial Cells

Thijs Pasman, Danielle Baptista, Sander van Riet, Roman K. Truckenmüller, Pieter S. Hiemstra, Robbert J. Rottier, Naomi M. Hamelmann, Jos M. J. Paulusse, Dimitrios Stamatialis and André A. Poot

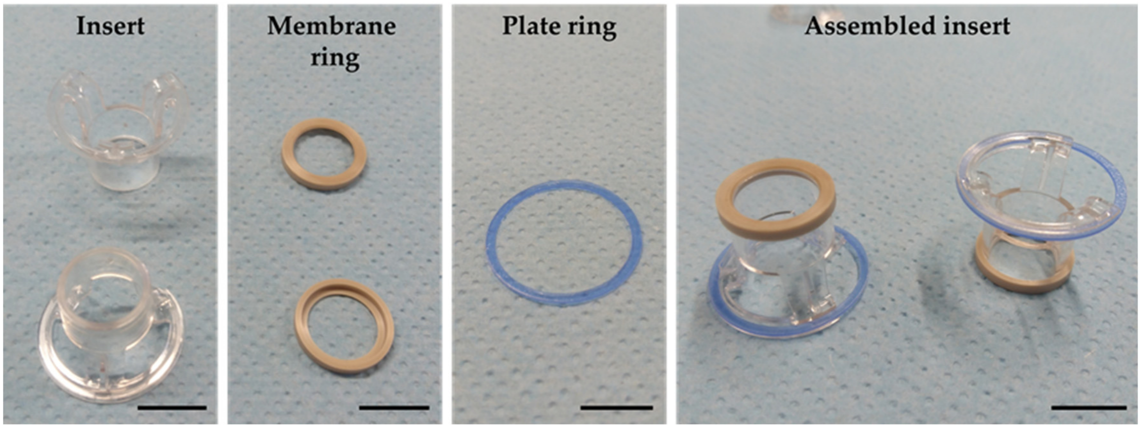

Figure S1. Cell culture inserts for the poly(trimethylene carbonate) (PTMC) membranes. Depicted are, from left to right: commercial Transwell® cell inserts with the standard poly(ethylene terephthalate) (PET) membrane removed, custom made membrane and plate rings (made from poly(ether ether ketone) (PEEK) and poly(propylene) (PP), respectively), and the assembled insert (insert with membrane and plate rings). PTMC membranes were secured to the bottom of the inserts by use of the membrane rings. The plate rings were situated between the top of the culture plate wells and the inserts, raising the latter to allow cell culture medium to reach the underside of the inserts. Scale bar: 1 cm.
